# Phosphorylated α-synuclein in Parkinson’s disease: correlation depends on disease severity

**DOI:** 10.1186/s40478-015-0185-3

**Published:** 2015-01-31

**Authors:** Tessandra Stewart, Vesna Sossi, Jan O Aasly, Zbigniew K Wszolek, Ryan J Uitti, Kazuko Hasegawa, Teruo Yokoyama, Cyrus P Zabetian, James B Leverenz, Alexander Jon Stoessl, Yu Wang, Carmen Ginghina, Changqin Liu, Kevin C Cain, Peggy Auinger, Un Jung Kang, Poul Henning Jensen, Min Shi, Jing Zhang

**Affiliations:** Department of Pathology, University of Washington School of Medicine, 325 9th Avenue, HMC Box 359635, Seattle, WA 98104 USA; Department of Physics and Astronomy, University of British Columbia, Vancouver Hospital and Health Sciences Centre, Vancouver, BC Canada; Department of Neurology, St. Olavs Hospital, Trondheim, Norway; Department of Neurology, Mayo Clinic Florida, Jacksonville, FL USA; Department of Neurology, National Hospital Organization, Sagamihara National Hospital, Kanagawa, Japan; Geriatric Research, Education and Clinical Center, Veterans Affairs Puget Sound Health Care System, Seattle, WA USA; Parkinson’s Disease Research, Education and Clinical Center, Veterans Affairs Puget Sound Health Care System, Seattle, WA USA; Department of Neurology, University of Washington School of Medicine, Seattle, WA USA; Department of Psychiatry and Behavioral Sciences, University of Washington School of Medicine, Seattle, WA USA; Mental Illness Research, Education and Clinical Center, Veterans Affairs Puget Sound Health Care System, Seattle, WA USA; Pacific Parkinson’s Research Centre, University of British Columbia and Vancouver Coastal Health, Vancouver, BC Canada; Department of Neurosurgery, Tongji Hospital, Tongji Medical College, Huazhong University of Science and Technology, Wuhan, Hubei 430030 China; Department of Endocrinology and Metabolism and Xiamen Diabetes Institute, the First Affiliated Hospital of Xiamen University, Xiamen, China; Department of Biostatistics, University of Washington School of Public Health, Seattle, WA USA; Department of Neurology, Center for Human Experimental Therapeutics, University of Rochester School of Medicine and Dentistry, Rochester, NY USA; Department of Neurology, University of Chicago Medicine and Biological Sciences, Chicago, IL USA; Department of Biomedicine, Aarhus University, Ole Worms alle 1170, DK-8000 Aarhus-C, Denmark

**Keywords:** Parkinson’s disease, Cerebrospinal fluid, Biomarker, α-synuclein, Phosphorylation, DATATOP

## Abstract

**Introduction:**

α-Synuclein (α-syn) is a key protein in Parkinson’s disease (PD), and one of its phosphorylated forms, pS129, is higher in PD patients than healthy controls. However, few studies have examined its levels in longitudinally collected cerebrospinal fluid (CSF) or in preclinical cases. In this study, CSF and clinical data were contributed by >300 subjects from three cohorts (the longitudinal DATATOP cohort, a large cross-sectional cohort, and a cohort of *LRRK2* mutation carriers).

**Results:**

Consistent with our previous observation that CSF pS129 positively correlated with Unified Parkinson’s Disease Rating Scale (UPDRS) scores, CSF pS129 in the DATATOP cohort increased over approximately two years of disease progression (mean change 5.60 pg/ml, p = 0.050). Intriguingly, in the DATATOP cohort, pS129 negatively correlated with UPDRS scores at the baseline (R = −0.244, p = 0.017), but not final point, suggesting that this association may depend on disease stage. Reanalysis of our previous cohort with stratification by PD stage, and addition of a cohort of *LRRK2* mutation carriers with very early/preclinical PD, supported the idea that the relationship between CSF pS129 and disease severity over a wider range of PD stages might be represented with a U-shaped curve, in which lower pS129 levels correlated with worse clinical condition at early stages, but better condition at later stages.

**Conclusion:**

The observation of a negative-to-positive transition of correlation of pS129 to disease severity as PD progresses could have profound impact on how pS129 is used as a biomarker clinically as well as in modeling PD experimentally.

## Introduction

Parkinson’s disease (PD) is a common age-related neurodegenerative disease, with a complicated etiology featuring both genetic and environmental components [1,2]. The abundant, membrane-associated protein α-synuclein (α-syn) is the primary component of Lewy bodies (LBs), a defining feature of PD, although whether these inclusions are themselves detrimental, or are a protective mechanism for sequestering toxic soluble oligomeric forms of α-syn, remains controversial [3,4].

Posttranslational modifications of α-syn, particularly phosphorylation at serine 129 (pS129), may be critical in PD [5,6], as α-syn in LBs from patients with sporadic [5-8] and genetic [9] forms of PD is overwhelmingly phosphorylated at S129. Further, pS129 alters characteristics of α-syn such as its propensity for aggregation [6,10,11], toxicity [10-12], and protein associations [13,14]. However, considerable controversy exists over the effects of pS129 on neurodegeneration in PD, with contradictory findings in transgenic fly [10] and mammalian models studying effects of phosphorylation on α-syn toxicity [11,15] and neurodegeneration [16]. These contrasts suggest that the effects of pS129 may be highly model-dependent, making assessment of the natural course of pS129 in human disease imperative for more accurate modeling of human PD, as well as to assist future translational investigations.

Our recent study in a cross-sectional cohort found that, unlike total α-syn, CSF pS129 is higher in PD than healthy controls [17]. Remarkably, it also significantly correlated with PD severity, suggesting that, in addition to being mechanistically important in the pathology of PD, pS129 may serve as a biomarker for PD progression. However, this hypothesis must be interrogated in longitudinally collected samples, particularly in subjects without confounding factors presented by medication. Yet, longitudinal studies of PD face a number of challenges, including repeated collections of CSF over a prolonged time, allowing enough time to detect biochemical alterations in this slowly progressive disease.

We studied pS129 in the DATATOP (the deprenyl and tocopherol antioxidative therapy of Parkinsonism) cohort, in which recently diagnosed PD patients (that is, subjects with early, unmedicated clinical PD) underwent repeated CSF sampling, two years apart. To extend our findings to a wider range of disease stages, we further examined CSF samples obtained from subjects with *LRRK2* mutations (which lead to late-onset PD similar to the idiopathic disease), including patients at preclinical stages [18,19], and results published previously [17,20] from a large multi-center collaborative cohort including subjects with later stage PD. With these cohorts, we identified a potential non-linear relationship between CSF pS129 and disease severity.

## Materials and methods

### Study subjects

Cohorts used in this study were approved by the Institutional Review Boards of all participating institutions. Although cohorts were recruited and samples were collected independently, similar protocols were used throughout, including collection between 6 and 10 am, freezing immediately after collection, use of polypropylene tubes, addition of protease inhibitor cocktail, and thawing immediately prior to the assay. Samples were centrifuged for 10 minutes at 15,000 rpm before all assays (α-syn, pS129, or hemoglobin). Two longitudinal samples were collected from the DATATOP cohort subjects, and one cross-sectional sample was collected from each Multi-center collaborative cohort and *LRRK2* cohort subject.

#### DATATOP cohort

Detailed description of the DATATOP cohort, recruited in 1987, has been previously published, [21] as have the additional criteria used in our analyses [20,22]. To eliminate potential confounding effects, only those assigned to the placebo group were included in this study. Subjects were followed for ~24 months, with the primary outcome of time to “endpoint,” defined as the development of symptoms severe enough to require treatment with levodopa, as determined by a blinded clinician. Clinical data, such as Unified Parkinson’s Disease Rating Scale (UPDRS) score, was evaluated approximately every three months, and CSF was collected twice, once each at the baseline and final time points. We included subjects whose follow-up time was >6 months, resulting in a cohort of 95 PD patients.

#### Multi-center collaborative cohort

CSF samples were collected from a total of 209 PD patients, recruited at nine collaborative centers, using similar collection and quality control protocols. Patients underwent extensive clinical evaluation; a detailed description of this cohort, including pS129 scores, has been previously published [17]. Of note, the range of MMSE scores is large, and some of our subjects, especially those enrolled in Multicenter-collaborative cohort, have MMSE scores meeting the criteria of cognitive impairment or even dementia. However, as >75% typical PD patients show cognitive dysfunction eventually [23,24], we did not exclude these subjects, because it would be impossible to also exclude the equivalent patients (that is, those who would eventually become demented) from the cohorts with subjects at earlier stages.

#### LRRK2 cohort

CSF samples from 23 asymptomatic and seven symptomatic *LRRK2* mutation carriers at early stages (subjects with UPDRS > 20 were excluded to allow exclusive focus on early PD) were assessed. Note that disease duration, used to define “early” PD in the other cohorts, is not available, as most of these subjects have not yet been diagnosed with PD. Further details concerning recruitment, demographics, and other CSF markers in a subset of this cohort have been previously published [18,19].

### PET studies

Within one year of CSF sample collection, each *LRRK2* cohort subject traveled to the Pacific Parkinson’s Research Centre (Vancouver, BC, Canada) for positron emission tomography (PET) scans. Any antiparkinsonian medications were stopped at least 12 h before assessment. Subjects were scanned with ^11^C-(±)-α-dihydrotetrabenazine (TBZ), a ligand of vesicular monoamine transporter 2, as previously described [18,19]. All PET data were normalized to age-matched control values. Because the asymptomatic group included both subjects with marked reductions in TBZ scores (likely at an early PD stage) and subjects with normal TBZ scores (who may be truly unaffected at the time of sampling), we limited some analyses to subjects with age-normalized TBZ scores <1.0.

### Luminex assays

CSF pS129 was measured according to our previously published protocol [17]. Briefly, MicroPlex microspheres (Luminex, Austin, TX, USA) were coupled with anti-α-synuclein antibody (ASY-1 [25,26]). For each subject, 75 μl CSF was diluted with 25 μl assay diluent (0 · 1% bovine serum albumin [Sigma, St Louis, MO] in PBS). Analytes were detected using biotinylated anti-human pS129 antibody [27] and streptavidin-R-PE (Prozyme, Hayward, CA, USA). Plates were read on a LiquiChip Luminex 200 Workstation (Qiagen, Valencia, CA, USA). CSF α-syn was measured as previously described; [28] results for the entire DATATOP cohort have been published separately [20]. In all assays, samples were randomly distributed across plates to avoid confounding due to the plate location, and assay operators were blind to the disease status or time point of samples.

Although frozen DATATOP CSF samples underwent prolonged storage, concentrations of CSF markers (Aβ, tau, α-syn), reported elsewhere [20,22], were comparable to recently collected samples in our previous studies [28,29].

### Hemoglobin measurements

Blood contamination was assessed using Human Hemoglobin ELISA Quantitation Kit (Bethyl Lab Inc., Montgomery, TX, USA) according to the manufacturer’s instructions.

### Statistical analysis

All analyses were performed using PASW Statistics 19 (IBM, Chicago, IL). Our α-syn and pS129 assays typically result in inter-plate variability <15% and intra-plate variability <10% [17,28]; no corrections were made for assays that performed within this range. Within each cohort, interassay variability was ensured by including identical reference samples on each assay plate, and inter-plate variability controlled by correcting for these references. No corrections were made between cohorts. Values are reported as mean ± standard deviation (SD) unless otherwise noted. Change in biomarker levels was analyzed using a paired t-test on the difference (final-baseline) of CSF sample values following Shapiro-Wilk test for normality. Because a previous study demonstrated that blood contamination influences levels of total α-syn [28], 25 subjects with at least 1 sample containing >200 ng/ml hemoglobin, the level at which blood α-syn confounds CSF α-syn levels, were excluded from analysis comparing longitudinal changes in pS129, α-syn or pS129/α-syn ratio. Associations between CSF levels and UPDRS scores are reported as Pearson correlation. Association with age-normalized TBZ scores is reported as Spearman correlation, due to the highly non-normal distribution of TBZ scores. Subjects diagnosed with PD ≤2 years before sample collection (the median disease duration for DATATOP subjects at baseline) were considered “early” stage cases for the purpose of segregation of cohorts into “early” vs “late” PD.

## Results

### Demographic distribution of DATATOP cohort

Clinical evaluations and CSF were assessed in 95 subjects assigned to the original DATATOP placebo group. Demographic characteristics, clinical scores, and CSF levels of protein markers of all cohorts are presented in Table 1. Endpoint, defined as development of symptoms of sufficient severity to require administration of dopamine supplementing drugs, was reached by 72 DATATOP subjects.

**Table 1:** **Demographic characteristics of cohorts**

	**Baseline (N = 95)**	**Final (N = 95)**	**UW-collaborative (N = 209)**	**LRRK2 (N = 30)**
Age, y (mean ± SD)	61.08 ± 8.91	63.08 ± 8.68	65.94 ± 10.54	53.13 ± 13.88
Sex F/M (% Male)	35/60 (63%)	35/60 (63%)	52/157 (75%)	15/15 (50%)
Duration of disease, y				
Mean ± SD	1.9 ± 1.41	4.02 ± 1.55	7.9 ± 6.48	--
Range	0 – 6	1 – 9	0 – 42	
MMSE				
Mean ± SD	28.85 ± 1.57	28.81 ± 1.48	28.47 ± 3.07	--
Range	23 – 30	22 – 30	11 – 30	
H&Y^†^				
Median	2.0	2.0	2.0	--
Range	1.0 – 2.5	1.0 – 4.0	0.0 – 5.0	
UPDRS^‡^ Total				
Mean ± SD	24.87 ± 12.50	40.73 ± 16.63	--	--
Range	0 – 61	9 – 88		
UPDRS Motor				
Mean ± SD	16.90 ± 9.52	27.36 ± 12.13	23.72 ± 11.87	5.20 ± 4.73
Range	0 – 50	5 – 62	0 – 71	0 – 20
Ps129 (pg/ml)				
Mean ± SD	114.66 ± 17.14	117.89 ± 17.92	74.01 ± 26.67	63.79 ± 22.73
Range	57.11 – 151.63	68.66 – 170.78	0.00 – 203.17	35.2 – 145.1
Total α-syn (pg/ml)				
Mean ± SD	630.83 ± 703.08	639.48 ± 677.95	553.61 ± 409.22	744.78 ± 1169.94
Range	186.3 – 6343.5	173.6 – 4523.0	140.0 – 2990.0	170.98 – 5735.08
Ratio				
Mean ± SD	0.2530 ± 0.1232	0.2688 ± 0.1433	0.177 ± 0.098	0.172 ± 0.132
Range	0.01 – 0.77	0.02 – 0.86	0.00 – 0.53	0.01 – 0.56

^†^Hoehn and Yahr.

^‡^Unified Parkinson’s Disease Rating Scale3.

### Longitudinal alteration of phosphorylated α-syn in CSF

**Figure 1:**
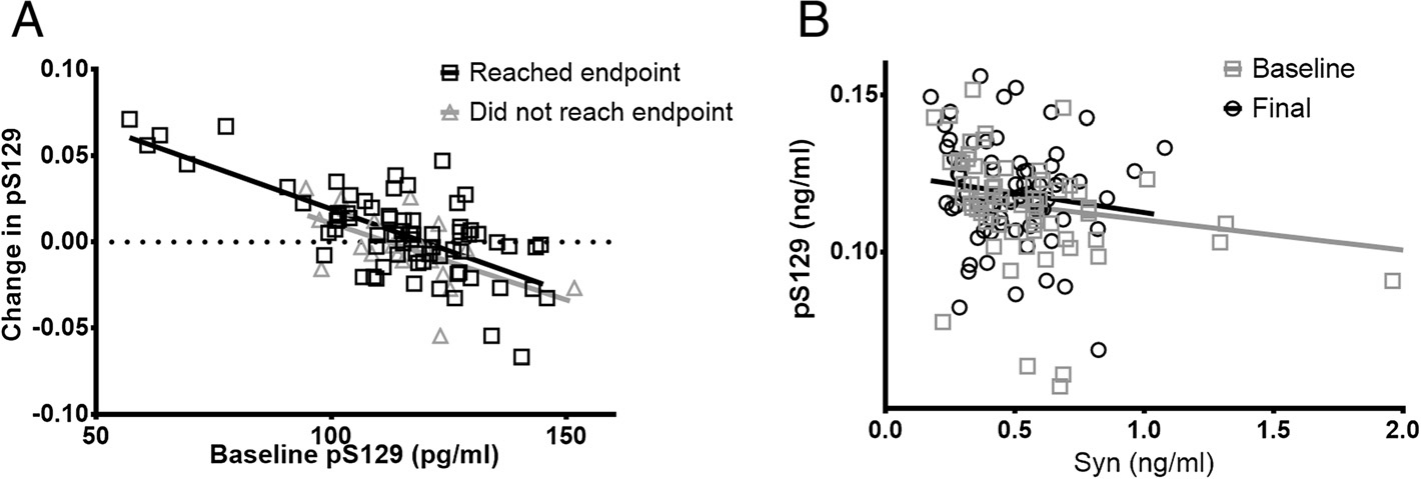
**Longitudinal changes in pS129 and relationship with total α-syn. A)** Change in pS129 by baseline level. Triangles: subjects that did not reach endpoint. Squares: subjects that reached endpoint. Solid line: regression line generated from all subjects combined. **B)** Association between total α-syn and pS129 levels by time point. Circles: baseline time point. Squares: final time point. Lines represent correlation between total α-syn and pS129 for each time point.

We tested the change in pS129 and the pS129/α-syn ratio over the two-year DATATOP follow-up period. Despite a negative trend, suggesting a small decrease, no significant difference was observed between baseline and final total α-syn (mean difference −190.50 pg/ml, 95% CI −382.57, 1.56, p = 0.052), in subjects meeting Hgb cutoff. In contrast, both pS129 and pS129/α-syn ratio increased (5.60 pg/ml, 95% CI [−0.01, 11.21], p = 0.050 and 0.034; 95% CI [0.007, 0.061], p = 0.014, respectively). We examined separately the subset of patients who reached endpoint, and found that the differences between final and baseline scores for pS129 and pS129/α-syn ratio were somewhat larger, indicating that the changes are more pronounced in the subset of subjects that progressed to requiring levodopa therapy (Table 2; Figure 1). Examining the relationship between total and phosphorylated α-syn, we found that at baseline, pS129 was significantly correlated with α-syn (R = −0.420; p < 0.001), but not at the final time point (R = −0.109; p = 0.335).

**Table 2:** **Paired t-test, change in marker: (Hgb > 200 excluded for both α-syn and pS129 to allow direct comparison of identical subject groups**

	**Subjects reaching endpoint (N = 51)**			**Subjects not reaching endpoint (N = 19)**			**All subjects w/Hgb ≤ 200 (N = 70)**		
	**Mean**	**ST dev**	**Sig**	**Mean**	**ST dev**	**Sig**	**Mean**	**ST dev**	**Sig**
Ps129 (pg/ml)	**8.73**	**24.43**	**0.014**	−2.78	19.09	0.534	**5.60**	**23.53**	**0.050**
Ps129/Syn	**0.040**	**0.123**	**0.024**	0.018	0.079	0.347	**0.034**	**0.113**	**0.014**
Syn (pg/ml)	−169.93	877.37	0.173	−245.73	134.65	0.085	−190.50	805.51	0.052

Bold font indicates significance at the p=0.05 level.

This finding of increasing pS129 is quite consistent with previous studies; however, the DATATOP cohort does not include neurologically normal control subjects in which to examine age-related changes in CSF pS129, so any changes due only to aging would not be detected in this set. In order to approximate this measurement, we studied the cross-sectional relationship between age and pS129 at the baseline and final time points, and found no correlation (p > 0.05; Pearson correlation), in either the full cohort or only the subset that reached endpoint. In contrast to a previous study [28], there was no significant correlation between age and total α-syn at baseline (R = 0.176; p = 0.107); however, a significant correlation was observed between age and total α-syn at the final time point (R = 0.267; p = 0.017).

### CSF pS129 and disease severity in cohorts at differing stages of disease progression

Having observed a longitudinal increase in pS129 in recently diagnosed PD patients, we next sought to examine the association between CSF pS129 and disease severity across a wider range of PD stages than has previously been examined, by studying early clinical PD in recently diagnosed subjects (DATATOP), moderate to severe clinical PD (multi-center collaborative cohort) and very early/preclinical PD (*LRRK2* cohort).

#### DATATOP cohort: early clinical PD

We examined the relationship of CSF pS129 with PD severity. A negative correlation was observed between pS129 and UPDRS motor scores at baseline (R = −0.244, p = 0.017), but not at the final time point (Table 3), after approximately two years of disease progression, when motor scores were significantly worse. The pS129/α-syn ratio was not correlated with UPDRS scores at any time point. Controlling for age and gender did not significantly alter the results in any case (not shown).

**Table 3:** **Comparison of correlation between UPDRS motor scores and CSF pS129 levels, overall and stratified by disease duration**

		**DATATOP baseline**			**DATATOP final**			**Multi-center collaborative**			**LRRK2**		
		**N**	**Corr**	**P**	**N**	**Corr**	**P**	**N**	**Corr**	**P**	**N**	**Corr**	**P**
pS129	Whole cohort	**95**	**−0.244**	**0.017**	95	0.147	0.834	**199**	**0.147**	**0.038**	30	−0.050	0.794
	Disease duration ≤2 years	**69**	**−0.289**	**0.016**	18	−0.093	0.712	32	0.043	0.817	--		
	Disease duration >2 years	23	−0.067	0.778	74	−0.050	0.674	**164**	**0.173**	**0.027**	--		

B: baseline time point. F: final time point. Most LRRK2 cohort subjects have not yet received PD diagnoses. Disease durations not available for 3 DATATOP subjects and 3 multi-center collaborative cohort subjects.

Bold font indicates significance at the p=0.05 level.

The cross-sectional correlation with UPDRS (lower in subjects with worse UPDRS scores at baseline) and the longitudinal change in pS129 levels (increasing with progression of disease severity) appear contradictory. Additionally, the DATATOP baseline correlation results seemed to conflict with our previous study [17], in which pS129 was significantly positively correlated with UPDRS motor score in a large cohort. This apparent paradox could be explained if the negative correlation is unique to the baseline DATATOP cohort, rather than a feature of PD progression; however, we wished to investigate whether it might be a feature of a multi-stage response, reflecting a non-linear (U-shaped) time course of pS129 expression. Because a second, comparable longitudinal cohort was not available to study this question, we instead examined two additional cross-sectional cohorts, one with more advanced PD subjects [17] and one with preclinical/very early PD subjects [18,19], to attempt to estimate the course of CSF pS129 over a longer period of PD progression.

#### Comparison of DATATOP and multi-center collaborative cohorts: early vs moderate/severe clinical PD

**Figure 2:**
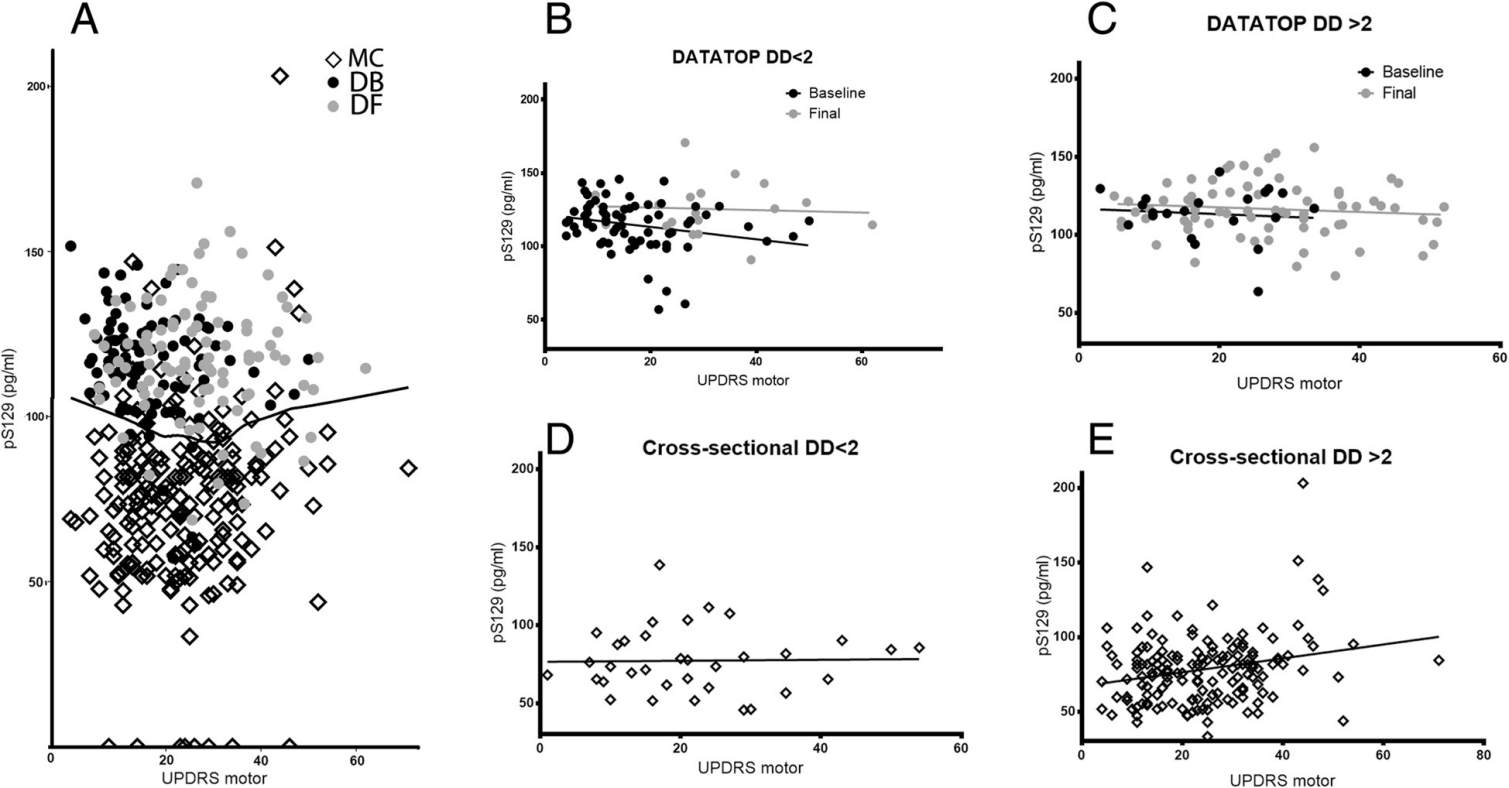
**The relationship between pS129 and motor symptoms changes with disease severity.** pS129 and UPDRS motor scores by disease duration **A)** Relationship between CSF pS129 and UPDRS motor scores in subjects in DATATOP and cross-sectional cohorts. Line is Loess curve for combined datasets including multi-center collaborative cohort and DATATOP baseline and final time points. **B)-E)** Relationship between pS129 and UPDRS in cohort subsets divided by disease stage. **B)** DATATOP DD less than 2 years. **C)** DATATOP DD greater than 2 years. **D)** Cross-sectional DD less than 2 years. **D)** Cross-sectional DD greater than 2 years. MC: Multi-center collaborative cohort. DD: Disease duration, time since diagnosis in years. DB: DATATOP baseline time point. DF: DATATOP final time point. Diamonds: multi-center collaborative cohort. Black circles: DATATOP baseline. Gray circles: DATATOP final.

To determine whether the difference in the direction of correlation between CSF pS129 and UPDRS motor scores in DATATOP and our previous multi-center collaborative cohort could be explained by the much earlier disease status of the DATATOP cohort, we re-analyzed both cohorts after stratifying by disease stage (Table 3; Figure 2 B-E), defining subjects with a disease duration less than or equal to the median for the baseline DATATOP cohort (2 years) as having early stage PD. In the DATATOP cohort, the significant negative correlation between UPDRS and pS129 was observed in the early stage subjects at the baseline time point (disease duration ≤ 2 years; n = 69), but not in the smaller group with early PD at the final time point (n = 18). No significant trend was observed at either time point in the subset with more advanced PD (disease duration >2 years; n = 23 and 74 for baseline and final, respectively). Next, we stratified the multi-center collaborative cohort using the same criteria, although this group included many more subjects with longer disease durations. The trend of increasing UPDRS motor scores with higher pS129 CSF values was significant in both the whole group (n = 199) and late stage PD subset (n = 164), but not in those with earlier stage PD. When the DATATOP baseline and final time point data (early PD) were analyzed together with the multi-center cross-sectional data (moderate to late PD), a U-shaped relationship between CSF pS129 and UPDRS motor scores appeared, i.e., a negative association during early PD (higher pS129 was associated with lower UPDRS and lesser disease severity), followed by an increasingly positive association with progressing disease (Figure 2A). Therefore, this data supports the idea that the direction of the association between pS129 and UPDRS is non-linear and dependent on disease stage, and there may be a transition between negative and positive correlation between CSF pS129 and disease severity, leading to a U-shaped curve over long periods of follow-up or a wide range of severities included in a cross-sectional study.

#### LRRK2 cohort: very early and preclinical PD

**Figure 3:**
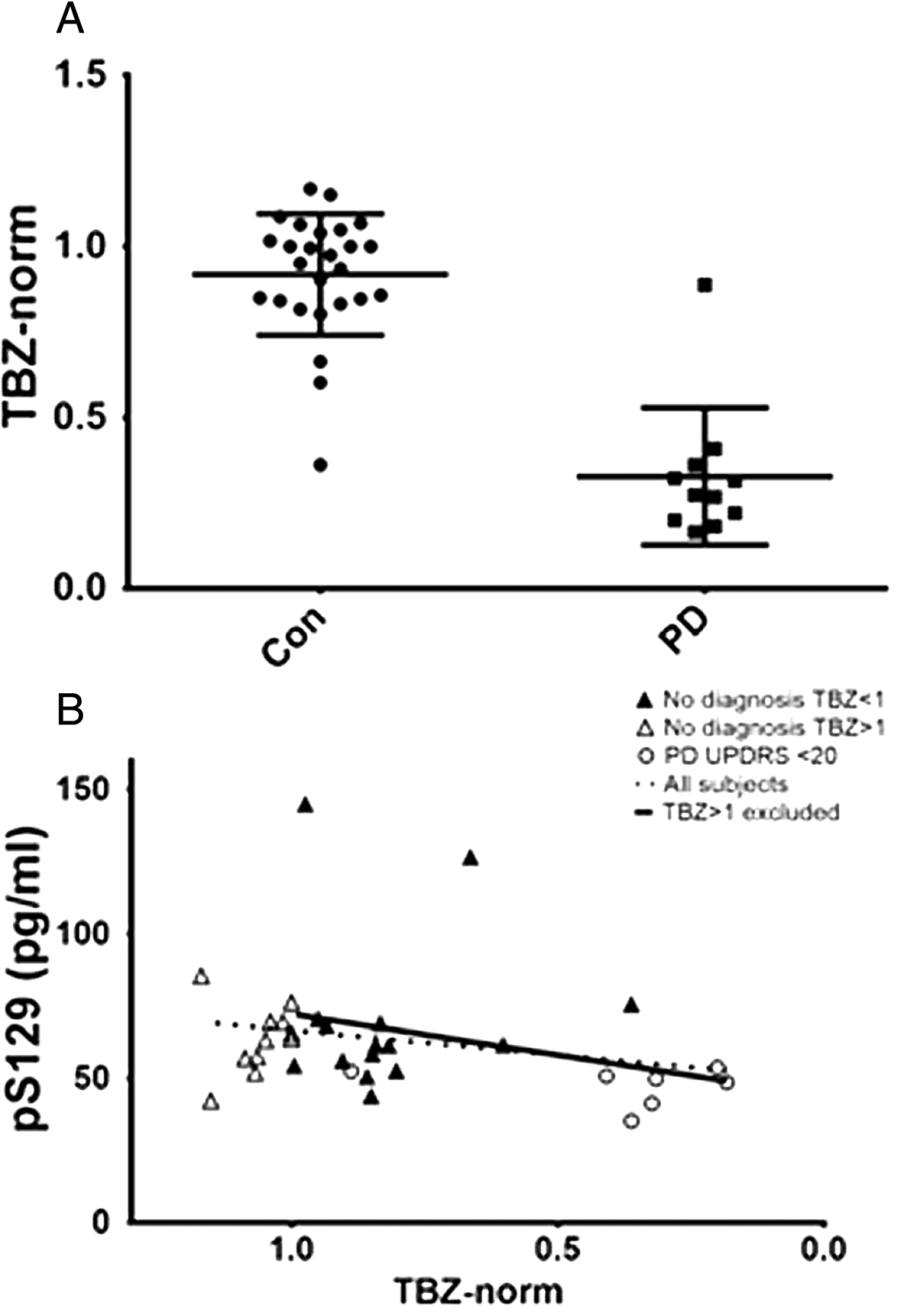
**LRRK2 subjects with greater brain pathology have lower CSF pS129. A)** Significant decrease in TBZ scores in subjects with PD diagnoses. **B**. Relationship between TBZ scores and pS129 in *LRRK2* mutation carriers. Note that x-axis is inverted to facilitate comparison with UPDRS measurements in Figure 2. Dotted regression lines represent whole cohort; solid lines represent cohort excluding subjects with TBZ scores ≥1. Black triangles: Subjects without PD diagnosis and TBZ < 1. Open triangles: Subjects with no diagnosis and TBZ > 1. Circles: Subjects with PD diagnosis and UPDRS motor scores < 20.

If the hypothesis that the correlation between CSF pS129 and disease severity is dependent on PD progression is true, then it would be expected that subjects with very early or preclinical PD would show a negative correlation (similar to DATATOP baseline). However, preclinical/very early PD patients have very low (or 0) UPDRS scores, making analysis of the correlation difficult. Therefore, we sought to test this hypothesis using a cohort of *LRRK2* mutation carriers, in which preclinical PD signs were assessed by neuroimaging (Table 1). As expected, no correlation was observed between pS129 and UPDRS (Table 3) in this small cohort of mostly clinically asymptomatic subjects, as the UPDRS values for asymptomatic subjects were near or equal to 0. However, *LRRK2* subjects underwent PET imaging, and those with clinical PD show markedly reduced age-normalized TBZ scores (Figure 3A); therefore, TBZ scores, which appear to undergo changes at an earlier stage than UPDRS, were used to measure severity of the pathology in these subjects. In *LRRK2* mutation carriers, a trend of decreased pS129 with greater disease severity was observed, and was significant in the subset of subjects with early PD pathology, indicated by TBZ < 1 (n = 20, Spearman’s rho 0.456, p = 0.043; Figure 3B). This result is consistent with the trend seen in early PD cases of DATATOP cohort, i.e. higher ps129 values were associated with higher TBZ scores and lesser disease severity (note that high TBZ scores and low UPDRS scores both reflect milder disease), suggesting that the finding of a negative relationship at early disease stages is not unique to the DATATOP cohort.

## Discussion

Several key findings are presented in this study: 1) pS129 and pS129/α-syn ratio increased in longitudinally collected CSF from untreated early PD patients, particularly those who progressed to requiring dopamine therapy; and 2) the relationship between pS129 and PD severity differed at earlier and later disease stages (high pS129 reflected less severe symptoms at earlier stages, but not at later stages). We examined this possibility using reanalysis of a previously studied cohort, after stratification by disease duration, and found that this progression appeared as a U-shaped curve when the two cohorts were combined. Moreover, this interpretation was supported by examination of a cohort of *LRRK2* carriers with early or preclinical PD.

### Longitudinal changes in pS129 in PD progressions

α-Syn in LBs is highly phosphorylated at S129 [5,6,10], particularly in more severe stages of Lewy pathology [7,20], suggesting a progressive increase in pS129 as PD advances. Additionally, cross-sectional studies in brain tissue and CSF indicate increased phosphorylated α-syn in PD [6,17], suggesting that it may be useful as a PD biomarker. However, whether it increases longitudinally in the CSF of individual subjects has been less well examined. Here, we found that pS129 increased over the approximately two-year DATATOP study, while α-syn, though it trended to be lower in more advanced stages, did not achieve statistical significance in the placebo group. The ratio of pS129/α-syn showed a greater increase, as anticipated given their opposite expected directions of change. This investigation is, to our knowledge, the first study of pS129 in living subjects with CSF collected longitudinally over a reasonably long period of time (about two years). The observation is entirely consistent with our previous cross-sectional study demonstrating that pS129 and the pS129/syn were higher in PD than in controls, and pS129 significantly positively correlated with severity of motor symptoms [17]. Our data, however, contrasted with another recent study of post-mortem ventricular fluid, which observed no difference in pS129 between PD patients and controls [30]. The lack of significance between advanced PD and controls, when assessed using ventricular fluids collected at autopsy, could be attributed to a number of factors, including cohort size, antibodies used, or use of autopsy ventricular fluid, which has a different protein composition from lumbar CSF [31]. However, later studies by the same group found increased plasma pS129 in PD, but no longitudinal increase in follow-up periods between 3 months and up to 4 years [32,33]. In light of our CSF results, the lack of further increase in plasma pS129 in longitudinal samples could be explained by a combination of different sample types, as well as by the follow-up period, particularly considering the modest magnitude of the change observed in CSF here, even over approximately two years. Based on our CSF investigations, it is likely that a much longer period of follow-up is needed to detect biochemical alterations in slow neurodegenerative processes [20,22].

### pS129 and disease severity: evidence for a non-linear pattern in PD progression?

The most intriguing observation in the current study is that pS129 negatively correlated with PD severity at baseline, i.e. at a relatively early stage of the disease, in the DATATOP cohort. We hypothesized that this paradoxical observation might depend on disease stage, and separation of two cohorts by stage showed a trend, in both cohorts, toward more positive correlation with increasing severity. The effect of this change would be reflected in clinical trials as contradictory associations between early and late cohorts, as was observed when comparing the baseline DATATOP and multi-center collaborative cohorts. We considered that this relationship could be explained if pS129 decreases in the initial or preclinical stages of PD (leading to the negative correlation at early but post-diagnosis time points such as DATATOP baseline), then progressively increases at later stages (leading first to a loss of any correlation, followed by the introduction of a positive correlation, as well as longitudinally observed increase in concentration). We therefore sought to determine if a non-linear relationship appeared when a wider range of disease stages are studied together, particularly by including subjects at very early stages. Remarkably, the trend of a negative correlation between pS129 and PD severity at early stages was maintained in a cohort consisting of *LRRK2* mutation carriers exhibiting early signs of PD. Although UPDRS scores were typically very low in these subjects, TBZ imaging, which reflects nigrostriatal damage robustly [34], showed that higher pS129 levels were associated with better disease states.

Of four groups (DATATOP baseline and final, multi-center collaborative, and *LRRK2*), only the DATATOP final cohort, at intermediate disease stage, showed no association between pS129 and UPDRS, in contrast to the late multi-center collaborative cohort, where a clear positive correlation is shown. This lack of correlation in a portion of DATATOP cohort may be explained by a mixture of the limited number of the early stage subjects at the final time point and the comparatively earlier status of even the “late” subjects, most of which just reached the point of requiring PD medication. This leads to the hypothesis that these subjects are at the stages where the pS129/severity relationship is undergoing a negative-to-positive transition (i.e., near the vertex of a U-shaped curve). It should also be noted that, although the reported severity (UPDRS scores) in the multi-center collaborative cohort overlapped substantially with that of the DATATOP final group, in the former, all subjects were on anti-parkinsonism medications, partially masking the true severity of their symptoms. In other words, subjects in this cohort can be considered to have more severe PD than DATATOP subjects with equivalent scores. For the reasons discussed above, it is not surprising that neither the levels of the markers at baseline, nor their changes over the duration of the study, predicted longitudinal progression in UPDRS of the DATATOP cohort (data not shown).

Taken together, these data suggest the possibility that the relationship between pS129 and disease severity may alter with progression. The mechanisms by which such a phenomenon occurs, and what it means for the role of pS129 in PD pathogenesis, remain to be examined. One possible interpretation of the current study is that phosphorylation increases as a compensatory mechanism, explaining both its increasing levels and apparently contradictory association with less severe symptoms at early-mid stages, but that the benefits are eventually overcome by accumulating negative effects of this or other PD-related changes, explaining how pS129 could continue to increase, and become associated with worse outcomes in patients with more advanced disease. Regardless of the mechanisms involved, however, these observations might provide some insight to the conflicting results obtained in various animal models of PD. For example, experiments in fly [10] and rat models [11,15] found opposite effects of phosphomimetic mutant α-syn on neuronal degeneration, and differing effects of phosphorylation on the propensity of α-syn to form aggregates have also been reported [6,10,11,16,35]. Further, differences have been observed between the phosphomimetic and genuine phosphorylated proteins [16,35], complicating interpretation of these studies. If alterations in pS129 depend on the stage of the disease, it is likely that other disease-related molecular interactions influence the effects of pS129 in the cell, and, for future studies, the effects of pS129 must be investigated in model systems that recapitulate the human condition as closely as possible. An important caveat that must be considered is that, while patients in the early/preclinical cohorts (DATATOP and *LRRK2*) were untreated, those in the multi-center collaborative cohort, in addition to being at more advanced stages, are also undergoing therapeutic treatment. With these datasets, one could argue that the differing relationship between disease severity and pS129 with progression is driven by effects of drug treatments (for example, treatment could result in increased pS129 levels, such that those with worse symptoms requiring increased therapy exhibit the highest levels, and masking a negative relationship such as was observed in both the DATATOP and *LRRK2* cohorts). While this remains a theoretical possibility, the fact that a further increase in pS129 occurred in the longitudinal DATATOP cohort (Table 2), where all subjects are unmedicated, indicates PD therapy is unlikely the primary cause of this observation.

### Additional considerations

Several additional caveats must also be considered. One obvious concern is the lack of neurologically normal controls in the DATATOP study, meaning that no group exists for comparison of PD-related changes in pS129 with its natural course with aging. Therefore, whether the increasing levels of pS129 observed in the DATATOP cohort are due to PD progression or aging cannot be definitively determined by this dataset alone. However, some data exists to suggest that aging is not the primary factor. First, we previously examined this question in our multi-center collaborative cohort, and found no relationship between age and pS129 in older PD patients or control subjects [31]. Further, we examined the cross-sectional relationship between pS129 and age in the DATATOP cohort, and again found no association. Together, these data suggest that CSF pS129 is not dependent on age in the older patients included here. However, this must be confirmed in longitudinally collected control subjects in later studies. Additionally, when using UPDRS to assess the utility of CSF pS129 and/or pS129/α-syn in monitoring the central nervous systems of PD patients, it is important to consider that biochemical and clinical measures of disease reflect different things: CSF α-syn and pS129 are measures of brain-wide pathology while UPDRS motor score reflects largely the degeneration of nigrostriatal system. Despite these caveats, sensitive and objective detection of alterations in CSF pS129 and total α-syn, which both alter over two years of progression, [20] would greatly aid clinical trials of novel, disease-modifying treatments targeting α-syn-related systems.

In summary, in this study of pS129 using longitudinal samples in addition to two cross-sectional cohorts, we provide evidence for pathological alterations of pS129, along with total α-syn, in the natural course of PD progression. These observations, though obtained in a large cohort, need to be further validated in an independent investigation, e.g. in prospective studies like the ongoing Parkinson’s Progression Markers Initiative. The significance of this study also goes beyond a biomarker research, informing future mechanistic studies of the disease, which should consider this natural course of pS129 in humans when modeling PD experimentally.

### Compliance with ethical standards

The authors report no conflicts of interest. Protocols including human subjects were approved by all participating institutions, and performed in accordance with the ethical standards of the institutional research committee and with the 1964 Helsinki declaration and its later amendments or comparable ethical standards. Informed consent was obtained from all subjects prior to any procedures.
